# The Role of Surveillance in the Association Between the Nurse Work Environment and Patient Safety Culture

**DOI:** 10.1002/nur.70056

**Published:** 2026-02-22

**Authors:** Kathryn A. Connell, Hyunmin Yu, Domenique Villani, Matthew D. McHugh, Clint Douglas

**Affiliations:** ^1^ Department of Biobehavioral Health Sciences University of Pennsylvania School of Nursing Philadelphia Pennsylvania USA; ^2^ Leonard Davis Institute of Health Economics University of Pennsylvania Philadelphia Pennsylvania USA; ^3^ Penn Medicine Pennsylvania Hospital Philadelphia Pennsylvania USA; ^4^ Center for Health Outcomes and Policy Research University of Pennsylvania School of Nursing Philadelphia Pennsylvania USA; ^5^ School of Nursing Queensland University of Technology Brisbane City Queensland Australia; ^6^ Office of Nursing and Midwifery Services Metro North Hospital and Health Service Herston Queensland Australia

## Abstract

Nursing surveillance has been identified as a core clinical process through which organizational conditions may influence patient safety; however, its explanatory role in the relationship between the nurse work environment and patient safety culture is not well established. This study examined whether nursing surveillance practices account for part of the association between the nurse work environment and patient safety culture in medical–surgical units. Cross‐sectional data were drawn from 1,597 medical–surgical nurses who participated in the RN4CAST‐Australia 2018 survey. The nurse work environment was measured using the Practice Environment Scale‐5, nursing surveillance using a validated 16‐item scale assessing the frequency of patient assessment practices for early detection of deterioration, and patient safety culture using a validated short form of the Hospital Survey on Patient Safety Culture. Structural equation modeling was used to estimate direct associations between the nurse work environment and patient safety culture and indirect associations via nursing surveillance. A more favorable work environment was directly associated with a more positive patient safety culture and more frequent surveillance practices. More frequent surveillance was also directly associated with a more favorable patient safety culture. In addition, the nurse work environment was indirectly associated with patient safety culture through increased surveillance. These findings suggest that nursing surveillance represents a specific, actionable mechanism linking work environment conditions to patient safety culture. Efforts to improve patient safety may therefore require both structural enhancements to the work environment and targeted strategies to strengthen proactive nursing surveillance.

## 1 Introduction

The nursing work environment is a well‐established determinant of patient safety outcomes (Al‐ghraiybah et al. 2024; Lake et al. 2019; Nascimento and Jesus 2020). Numerous studies have shown that favorable work environments, characterized by adequate staffing, supportive leadership, and effective interprofessional collaboration, are associated with a stronger safety culture, fewer adverse events, and lower mortality among hospitalized patients (Brubakk et al. 2021; Cho et al. 2016; Dutra and Guirardello 2021; Olds et al. 2017).

Despite consistent evidence of these associations, the mechanisms through which work conditions translate into safer clinical practice remain incompletely understood. Prior research has identified important pathways through psychosocial states (e.g., burnout, engagement) and care process deficits (e.g., missed or unfinished care) (Hall et al. 2016; Li et al. 2024; Liu et al. 2018; Spence Laschinger and Leiter 2006; Van Bogaert et al. 2014). However, nursing surveillance, defined as the continuous, purposeful acquisition, interpretation, and synthesis of patient data to detect early deterioration (Kelly and Vincent 2011), represents a proximal clinical process that has received comparatively limited attention as a potential mediator, despite its theoretical plausibility and clinical relevance.

Using structural equation modeling (SEM) in a large sample of medical‐surgical nurses, we examined whether surveillance partially explains the association between the work environment and patient safety culture. We hypothesized that favorable work environments would be positively associated with surveillance practices and patient safety culture; that surveillance practices would be positively associated with patient safety culture; and that surveillance practices would demonstrate a significant indirect effect linking the work environment to patient safety culture.

## 2 Background

### 2.1 Work Environment and Patient Safety

Across diverse hospital settings and international contexts, work environments characterized by adequate staffing levels, responsive nursing leadership, and collaborative interprofessional relationships consistently predict stronger safety cultures, lower rates of adverse events, improved nurse‐reported safety indicators, and reduced patient mortality (Al‐ghraiybah et al. 2024; Dutra and Guirardello 2021; Griffiths et al. 2019; Kirwan et al. 2013; Lake et al. 2019; Malinowska‐Lipień et al. 2021; Rochefort et al. 2020; Zaranko et al. 2023). These associations demonstrate remarkable consistency across studies and persist even after adjustment for potential confounding variables, establishing the work environment as a fundamental determinant of patient safety outcomes.

### 2.2 Mechanisms Linking Environment to Safety

Emerging research using mediation analysis helps disentangle how and why an exposure affects an outcome by testing whether the effect operates through an intermediate variable (the mediator), thereby beginning to clarify the pathways through which work conditions are associated with safety outcomes. Burnout and work engagement represent well‐documented mechanisms. Multiple systematic reviews and meta‐analyses have consistently demonstrated that burnout is associated with a compromised safety culture and increased adverse events in healthcare settings, including higher rates of nosocomial infections, patient falls, medication errors, and missed care or care left undone (Hall et al. 2016; Hodkinson et al. 2022; Li et al. 2024; Tawfik et al. 2019). Both burnout and work engagement are responsive to environmental factors such as staffing adequacy, leadership quality, and teamwork effectiveness (Lee et al. 2023; Murray et al. 2018). For example, Tawfik et al. (2023) administered a survey to 31 hospitals in the Midwestern region. They found that supportive leadership and adequate staffing were associated with lower odds of burnout and a concerning safety culture. Similarly, care process indicators, most notably missed care, have emerged as important mediators, with missed care increasing under poor work conditions and subsequently correlating with poor safety outcomes (He et al. 2025; Kalánková et al. 2020; Liu et al. 2018; Recio‐Saucedo et al. 2018).

This growing literature suggests multiple parallel pathways connecting work environment structure to safety outcomes through both worker psychological states and care delivery processes (Wee and Lai 2022). However, existing mediators either capture global occupational strain (e.g., burnout, engagement) or broad process failures (e.g., missed care), leaving specific safety‐critical clinical practices relatively underexplored as potential mechanisms for understanding these phenomena.

### 2.3 Theoretical Framework for Surveillance as a Mediator

Multiple theoretical frameworks are covered to position nursing surveillance as a potential mediator of environmental safety relationships. The Job Demands–Resources (JD‐R) model posits that environmental resources, such as adequate staffing, supportive leadership, and effective teamwork, sustain motivation and cognitive capacity. At the same time, excessive demands erode attentional resources (Demerouti et al. 2001). Within this framework, resource‐rich environments may plausibly support the sustained attention required for systematic clinical work, including nursing surveillance (Demerouti et al. 2001; Vander Elst et al. 2016).

Complementary perspectives from systems engineering and quality improvement further support this theoretical positioning. The Systems Engineering Initiative for Patient Safety (SEIPS) model explicitly conceptualizes how work system characteristics influence care processes, which, in turn, affect patient outcomes (Carayon et al. 2006). Donabedian's foundational structure‐process‐outcome framework posits that the structural features of healthcare delivery systems influence clinical processes, which, in turn, impact patient outcomes (Donabedian 2005). Viewed through these lenses, nursing surveillance functions as a resource‐enabled, demand‐sensitive clinical process through which structural work conditions are translated into safety‐relevant practices, ultimately leading to the establishment of shared safety norms (Holden et al. 2013; Peet et al. 2022).

### 2.4 Surveillance as a Safety‐Critical Process

Conceptually, nursing surveillance encompasses a vigilant, iterative cycle of focused patient assessment, pattern recognition, clinical interpretation, and timely escalation of concerns (Kelly and Vincent 2011). This process is closely linked to the prevention of patient deterioration and the reduction of failure‐to‐rescue events, which are primary indicators of nursing care quality (Doyon and Raymond 2024). Recent large‐scale randomized control trial evidence further underscores the importance of nursing surveillance, demonstrating that empowered nursing physical assessment ward practices can prevent rescue events and mortality among hospitalized adults (Douglas et al. 2024).

## 3 The Study

Unlike global process indicators such as missed care, surveillance captures moment‐to‐moment clinical work through which patient harm is actively prevented (Peet et al. 2019). For this study, we operationalize surveillance as the frequency of purposeful patient assessment and clinical interpretation activities that support timely clinical decision‐making, following the validated framework established by Douglas et al. (2016).

### 3.1 Study Hypotheses

Based on this theoretical foundation, we proposed the following hypotheses:

A more favorable nurse work environment is directly associated with a more positive patient safety culture.

A more favorable nurse work environment is directly associated with frequent nursing surveillance practices.

More frequent nursing surveillance practices are directly associated with a more positive patient safety culture.

A more favorable nurse work environment is indirectly associated with a more positive patient safety culture through more frequent nursing surveillance practices

To test these hypotheses, the study was designed to address a gap in the literature regarding the processes through which nurse work environments influence patient safety culture. While prior studies have demonstrated associations between work environments and safety outcomes, fewer have explicitly modeled discrete nursing care processes as explanatory mechanisms. Accordingly, this study uses a theory‐driven mediation framework to examine nursing surveillance as a hypothesized pathway linking work environment characteristics and patient safety culture.

## 4 Methods

### 4.1 Study Design and Data Source

This cross‐sectional, observational study utilized 2018 survey data from the RN4CAST‑Australia Study (McHugh et al. 2021), a representative investigation of nurses working in medical‑surgical units in Queensland. The RN4CAST‐Australia Study is part of the internationally recognized RN4CAST consortium, which has conducted a series of investigations evaluating the impact of nurse staffing, education, and work environments on both nurse and patient outcomes across multiple countries. Survey data were collected in 2018 from registered nurses and enrolled nurses in Queensland via email between May 1 and May 31, yielding a response rate of 27% (McHugh et al. 2021). Ethics approval was obtained from the Queensland University of Technology and the University of Pennsylvania.

### 4.2 Study Sample

Given the study's focus on the relationship among work environment, patient safety culture, and nurses' core surveillance practices, which was developed specifically for medical‐surgical unit nurses, the inclusion criteria were: (1) working in a hospital, (2) working as a direct care nurse, and (3) working in medical‐surgical units. Among the 7084 nurses who responded to the RN4CAST‐Australia survey in 2018, 18 who did not work in hospital settings were excluded. An additional 2622 nurses whose roles were not direct care were excluded, followed by 2847 nurses who did not work in medical‐surgical units. This resulted in an analytic sample of 1597 nurses.

### 4.3 Study Variable

#### 4.3.1 Independent Variable: Nurse Work Environment

The nurse work environment was measured using the validated 5‐item short form of the Practice Environment Scale of the Nursing Work Index (PES‐5) (Lake et al. 2024). The scale includes the following items: (1) administration that listens and responds to nurse concerns; (2) doctors and nurses have good working relationships; (3) a nurse manager who is a good manager and leader; (4) enough staff to get the work done; and (5) a clear philosophy of nursing that pervades the patient care environment. The PES‐5 demonstrated strong internal consistency, with a Cronbach's alpha exceeding 0.80. Construct validity was supported by expert panel evaluations, which ranked the selected items highly within their respective subscales. Criterion validity was established through analyses of variance comparing mean PES‐5 scores across responses to a single‐item measure of the work environment (Lake et al. 2024). Each item was scored on a 4‐point Likert scale ranging from 1 (strongly agree) to 4 (strongly disagree). For analysis, all items were reverse‐coded so that higher scores reflected more favorable work environments. The Cronbach's alpha was 0.79 in our sample.

#### 4.3.2 Mediating Variable: Core Surveillance Practices

Core surveillance practices were measured using the 16‐item Core Physical Assessment Skills for Patient Safety in Medical‐Surgical Units instrument, which identifies the set of physical assessment skills deemed essential for safe nursing surveillance in medical‐surgical units. These skills were established through a rigorous consensus process involving multiple rounds of Delphi surveys with expert medical‐surgical nurses and educators. They were validated as the core assessment activities necessary for detecting early signs of patient deterioration (Douglas et al. 2016). For each skill, a content validity index was calculated as the proportion of participants who assigned ratings of 4 (important) or 5 (extremely important). Consensus was defined a priori as at least 80% agreement for each item. The stability of the panel's response patterns across rounds was also evaluated to confirm that consensus had been achieved (Douglas et al. 2016).

In this study, nurses were asked: “How often do you monitor every patient assigned to you for early changes in their condition using the following physical assessment skills?” Responses were provided for each of the 16 skills using a 5‐point Likert scale, ranging from 1 (never) to 5 (always). Higher scores indicated more frequent engagement in core surveillance practices. The Cronbach's alpha was 0.91 in our sample.

#### 4.3.3 Dependent Variable: Patient Safety Culture

Patient safety culture was measured using seven items from the Hospital Survey on Patient Safety Culture (HSOPS), initially developed by the Agency for Healthcare Research and Quality (Sorra and Nieva 2004). To minimize respondent burden while still capturing a broad range of organizational features relevant to patient safety, we employed the validated 7‐item short form (Olds et al. 2017). The items included: (1) staff feel like their mistakes are held against them; (2) staff feel free to question the decisions or actions of those in authority; (3) we discuss ways to prevent errors from happening again; (4) important patient care information is often lost during shift changes or when another provider is covering my patients; (5) things “fall between the cracks” when transferring patients from one unit or care setting to another; (6) we are given feedback about changes put into place based on incident reports; and (7) the actions of management show that patient safety is a top priority.

This 7‐item scale demonstrated strong psychometric properties. Exploratory factor analysis identified a single factor (Eigenvalue = 2.56), with factor loadings ranging from 0.54 to 0.66. The scale showed robust internal consistency (Cronbach's alpha = 0.80), which decreased if any item was removed. Convergent validity was supported by item‐rest correlations ranging from 0.45 to 0.58 (Olds et al. 2017). Each item was rated on a 5‐point Likert scale ranging from 1 (strongly agree) to 5 (strongly disagree). The four positively worded items (2, 3, 6, and 7) were reverse‐coded so that higher scores indicated a more favorable patient safety culture. The Cronbach's alpha was 0.80 in our sample.

#### 4.3.4 Covariates

We included years of nursing experience, highest nursing degree, gender, and employment status as covariates in the model to account for individual‐level differences that may influence both assessments of the work environment and engagement in surveillance practices. Years of nursing experience were treated as a continuous variable, as more experienced nurses may demonstrate greater clinical expertise, confidence, and efficiency in conducting physical assessments (Kelly and Vincent 2011). Compared with the other study variables, years of nursing experience exhibited a wide distribution across the sample. To address potential scaling issues and facilitate model convergence, this variable was standardized prior to analysis. The highest nursing degree, operationalized as a binary variable (baccalaureate degree or higher vs. hospital program or diploma), was controlled for because educational preparation may affect surveillance practices. Gender (female vs. male) was included as a covariate given evidence that gendered experiences in nursing can affect role expectations, communication patterns, and workplace interactions, potentially influencing both work environment assessments and safety culture (Göktepe and Sarıköse 2022). Finally, employment status (full‐time vs. part‐time) was considered relevant because workload, exposure to the work environment, and integration into team dynamics may differ by employment status (Zeytinoglu et al. 2007), thereby shaping both surveillance practices and perceptions of patient safety culture.

### 4.4 Statistical Analysis

We utilized R version 4.5.1 to examine the relationships among work environment, core surveillance practices, and patient safety culture among nurses in the medical‐surgical unit. SEM was conducted using the *lavaan* package (Beaujean 2014; Rosseel 2012). A two‐stage modeling approach was applied (Cheung and Chan 2005). In the first stage, confirmatory factor analysis (CFA) was performed to evaluate the factorial validity of the latent constructs and the adequacy of the measurement model. The three latent constructs (work environment, core surveillance practices, and patient safety culture) were included in this stage. Modification indices were also reviewed to identify potential sources of model misfit and to guide re‐specification of the measurement model as appropriate (Schreiber et al. 2006). Residual correlations were introduced between items related to vital signs (e.g., blood pressure, heart rate, temperature) and between items related to breathing within the core surveillance practices scale, as nurses often assess these indicators concurrently. Model fit was then evaluated using multiple indices, including the chi‐square ( *χ* ^2^) test, the comparative fit index (CFI), the non‐normed fit index (NNFI), the root mean square error of approximation (RMSEA), and the standardized root mean square residual (SRMR). A non‐significant *χ*² test (*p* > 0.05) indicates good model fit; however, this test is highly sensitive to sample size. In large samples, even trivial discrepancies between the model and the data can result in statistically significant *χ*² values. Therefore, we prioritized the remaining fit indices (Shi et al. 2019). In accordance with established guidelines (Hu and Bentler 1999; Schumacker and Lomax 2004), good model fit was indicated by CFI and NNFI values exceeding 0.90 or 0.95, RMSEA values below 0.06, and SRMR values under 0.05. These fit indices served as key criteria for evaluating the adequacy of the hypothesized model in relation to the collected data.

**Figure 1:**
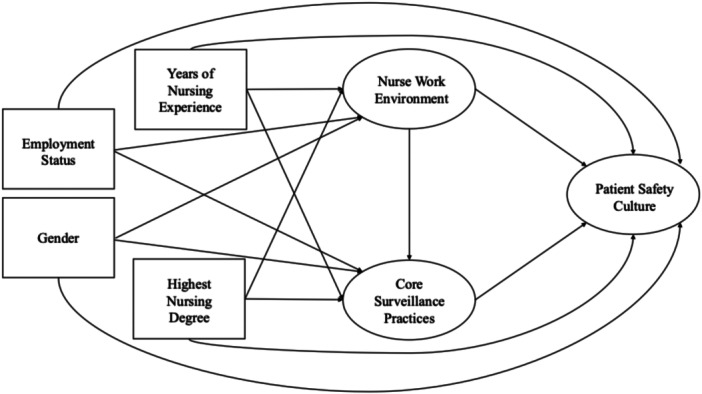
The hypothesized model included three latent variables: (1) Nurse work environment, (2) core surveillance practices, and (3) patient safety culture, as well as four observed variables: years of nursing experience, employment status, gender, and highest nursing degree.

In the second stage, SEM was conducted to assess whether the hypothesized model (Figure 1) demonstrated acceptable fit and to estimate the associations among variables. The SEM included three latent variables and four observed variables (years of nursing experience, highest nursing degree, gender, and employment status). The same fit indices were used to evaluate the structural equation model. A mediation analysis was then conducted to examine both the direct association between the nurse work environment and patient safety culture, as well as the indirect association through core surveillance practices. To enhance the robustness of the model, bootstrap standard errors were estimated using 1000 replicates.

The total sample (*N* = 1597) had a moderate proportion of missing data (28.6%), which was assumed to be missing at random (MAR) and addressed using full information maximum likelihood (FIML). Given the unknown mechanism underlying the missingness, this approach allowed us to utilize all available data without imputing missing values (Enders and Bandalos 2001).

## 5 Results

### 5.1 Sample Characteristics

Table 1 presents the characteristics of the 1597 nurses in the analytic sample. Participants had an average of 14.3 years of nursing experience (SD = 12.3). The sample was predominantly female (89.0%), and most nurses reported working part‐time (73.5%). The majority held a bachelor's degree or higher (87.6%), while 12.4% reported completing a hospital program or diploma.

**Table 1:** Nurse characteristics (*N* = 1597).

Characteristic	Value
Years of nursing experience, mean (SD)	14.3 (12.3)
Gender, *n* (%)^a^	
Female	1122 (89.0)
Male	139 (11.0)
Employment status, *n* (%)^a^	
Full time	422 (26.5)
Part time	1171 (73.5)
Highest nursing degree, *n* (%)^a^	
Hospital program or diploma	158 (12.4)
Bachelor's degree or graduate degree	1116 (87.6)

Abbreviation: SD, standard deviation.

^a^ The numbers do not add up to the total of 1597 due to missing values.

### 5.2 Measurement Model

Table 2 presents the results of the complete measurement model, which was estimated to evaluate the adequacy of the latent variable structure prior to testing the structural relationships. All observed indicators loaded significantly onto their hypothesized latent constructs, with standardized factor loadings ranging from 0.318 to 0.871 (all *p* < 0.001), indicating that the items were meaningful indicators of their respective factors.

**Table 2:** Measurement model.

Latent Factors	*n*	Items	Mean	SD	Standardized factor loadings
Work Environment (*α* = 0.79)	1406	Administration that listens and responds to nurse concerns.	2.5	0.9	0.669
	1413	A nurse manager who is a good manager and leader.	3.1	0.9	0.607
	1419	Doctors and nurses have good working relationships.	2.8	0.8	0.424
	1419	Enough staff to get the work done.	2.4	0.9	0.589
	1404	A clear philosophy of nursing that pervades the patient care environment.	3.0	0.7	0.524
Core physical assessment skills (*α* = 0.91)	1295	Assess airway patency	4.4	0.8	0.418
	1293	Measure respiratory rate	4.6	0.6	0.419
	1292	Evaluate work of breathing	4.5	0.7	0.480
	1281	Measure oxygen saturation	4.6	0.6	0.381
	1292	Palpate pulse rate and rhythm	4.2	0.9	0.480
	1291	Measure BP by auscultation	3.5	0.9	0.318
	1292	Assess urine output	4.0	0.8	0.474
	1290	Assess level of consciousness	4.7	0.5	0.366
	1291	Evaluate speech	4.3	0.9	0.499
	1286	Assess for pain	4.7	0.5	0.376
	1291	Measure body temperature	4.6	0.6	0.378
	1289	Inspect skin integrity	4.1	0.8	0.487
	1292	Inspect and palpate skin for signs of pressure injury	4.0	0.8	0.528
	1292	Observe any wounds, dressings or drains, invasive lines	4.4	0.7	0.467
	1288	Observe ability to transfer and mobilize	4.4	0.7	0.509
	1288	Assess bowel movements	4.2	0.8	0.513
Patient safety culture (*α* = 0.80)	1340	Staff feel like their mistakes are held against them.	2.9	1.0	0.541
	1335	Staff feel free to question the decisions or actions of those in authority.	2.9	1.1	0.716
	1337	We discuss ways to prevent errors from happening again.	3.8	0.9	0.673
	1334	Important patient care information is often lost during shift changes or when another provider is covering my patients.	2.8	1.0	0.388
	1335	Things “fall between the cracks” when transferring patients from one unit or care setting to another.	2.7	1.0	0.375
	1332	We are given feedback about changes put into place based on incident reports.	3.2	1.0	0.647
	1331	The actions of management show that patient safety is a top priority.	3.5	1.1	0.871

Abbreviation: SD, standard deviation.

Overall model fit statistics supported the adequacy of the hypothesized three‐factor measurement model. Although the *χ*² test was statistically significant (*χ*² = 1588.29, *df* = 328, *p* < 0.001), this result was expected given the large sample size; therefore, greater emphasis was placed on alternative fit indices. The CFI (0.926) and NNFI (0.915) exceeded the recommended threshold of 0.90, indicating good incremental fit. The RMSEA (0.052; 95% CI = 0.049–0.055) fell below the recommended cutoff of 0.06, and the SRMR (0.043) was below the recommended cutoff of 0.05. Collectively, these results support the construct validity of the measurement model and justify proceeding to the evaluation of the full structural equation model.

### 5.3 Hypothesis Testing: Structural Equation Model Results

The structural equation model was evaluated using multiple indices of model fit to assess how well the hypothesized relationships among nurse work environment, core surveillance practices, and patient safety culture were supported by the data. The *χ*² test was statistically significant ( *χ*² = 1794.84, df = 432, *p* < 0.001), as expected given the large sample size and the test's sensitivity to minor model‐data discrepancies. Therefore, additional fit indices were used to evaluate overall model adequacy.

**Figure 2:**
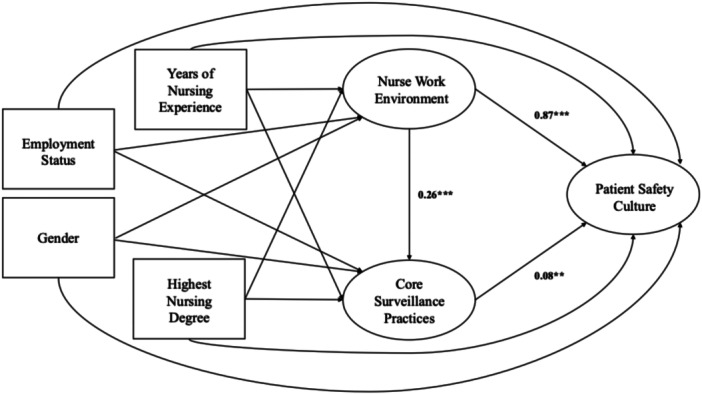
The final structural equation model examined the associations among three latent variables: (1) Nurse work environment, (2) core surveillance practices, and (3) patient safety culture, while accounting for observed covariates. The model demonstrated acceptable fit (CFI = 0.921; NNFI = 0.910; RMSEA = 0.044 [95% CI: 0.042–0.047]; SRMR = 0.044). This figure presents standardized coefficients for the direct associations. The indirect association (Work environment → Core surveillance practices → Patient safety culture) was also statistically significant (standardized *β̂* = 0.02, *p* = 0.001). *Note*: **p < 0.01, ***p < 0.001.

The full structural equation model (Figure 2) demonstrated good fit. Model fit indices met recommended thresholds: the CFI (0.921) and NNFI (0.910) exceeded the 0.90 criterion, the RMSEA (0.044; 95% CI = 0.042–0.047) was below the 0.06 cutoff, and the SRMR (0.044) was below the 0.05 threshold. These fit indices indicate that the structural equation model provides a good representation of the underlying relationships among the latent constructs, supporting the hypothesized pathways linking nurse work environments to patient safety through core surveillance practices. Results for each hypothesis (Table 3) are presented below.

**Table 3:** Results from structural equation modeling.

	Unstandardized *β̂*	SE	95% CI	Standardized *β̂*	*p*
Direct association					
Work environment → Patient safety culture	0.71	0.05	0.62–0.81	0.87	< 0.001
Work environment → Core surveillance practices	0.16	0.03	0.11–0.22	0.26	< 0.001
Core surveillance practices → Patient safety culture	0.11	0.03	0.04–0.17	0.08	0.001
Indirect association					
Work environment → Core surveillance practices → Patient safety culture	0.02	0.01	0.01–0.03	0.02	0.001

*Note:* The model controlled for years of nursing experience, highest nursing degree, gender, and employment status.

Abbreviations: CI, confidence interval; SE, standard error.

### 5.4 H1: A More Favorable Nurse Work Environment Is Directly Associated With a More Positive Patient Safety Culture

Consistent with H1, a more favorable nurse work environment was strongly and directly associated with a more positive patient safety culture (standardized *β̂* = 0.87, *p* < 0.001).

### 5.5 H2: A More Favorable Nurse Work Environment Is Directly Associated With Frequent Nursing Surveillance Practices

Supporting H2, the nurse work environment was significantly and directly associated with more frequent use of core surveillance practices (standardized *β̂* = 0.26, *p* < 0.001).

### 5.6 H3: More Frequent Nursing Surveillance Practices Are Directly Associated With a More Positive Patient Safety Culture

Results also supported H3. More frequent core surveillance practices were directly associated with a more positive patient safety culture (standardized *β̂* = 0.08, *p* = 0.001).

### 5.7 H4: A More Favorable Nurse Work Environment Is Indirectly Associated With a More Positive Patient Safety Culture Through Nursing Surveillance Practices

Consistent with H4, the nurse work environment demonstrated a statistically significant indirect association with patient safety culture through core surveillance practices (standardized *β̂* = 0.02, *p* = 0.001), indicating partial mediation.

### 5.8 Covariates

Among covariates, greater years of nursing experience were significantly associated with more frequent core surveillance practices (standardized *β̂* = 0.15, *p* < 0.001). No covariates were found to be significantly associated with patient safety culture.

## 6 Discussion

This study examined whether nursing surveillance explains part of the association between the nurse work environment and patient safety culture. It provides empirical evidence that nursing surveillance partially explains the relationship between the nurse work environment and patient safety culture. The structural equation model supported all hypothesized pathways. Favorable work environments were directly and strongly associated with positive safety culture, while also significantly enhancing frequent surveillance practices. Surveillance, in turn, was significantly associated with safety culture, creating a statistically significant indirect pathway that represents a meaningful mechanism linking structural conditions to safety outcomes.

### 6.1 Theoretical Integration

These findings align with and extend theoretical predictions from both the Job Demands‐Resources (JD‐R) model and systems engineering frameworks (Carayon et al. 2006; Demerouti et al. 2001; Donabedian 2005). While the JD‐R model does not explicitly explain surveillance behaviors, our findings extend this framework by identifying a plausible clinical process through which job resources may translate into safety‐relevant practices. The observed pattern also supports the structure‐process‐outcome logic central to both SEIPS and Donabedian frameworks, with surveillance occupying the critical process link between environmental structure and safety outcomes.

The modest magnitude of the surveillance‐mediated pathway, relative to the significant direct association with the work environment, warrants interpretation within the broader theoretical context. Patient safety culture reflects multiple organizational processes beyond surveillance, including communication patterns, incident reporting and learning systems, teamwork effectiveness, and leadership behaviors (Bisbey et al. 2021; Broetje et al. 2020). The present findings do not contradict the importance of these alternative pathways; instead, they isolate surveillance as one empirically supported mechanism while acknowledging that work environments influence safety culture through numerous parallel channels (Nahrgang et al. 2011).

### 6.2 Clinical and Organizational Implications

The identification of nursing surveillance as a mediator yields several actionable implications for improving patient safety. First, focused improvements in structural work environments, including adequate staffing levels, responsive nursing leadership, and enhanced interprofessional collaboration, are likely to enhance safety culture both directly and through enabling more proactive surveillance (Al‐ghraiybah et al. 2024). These findings provide additional justification for organizations to shift their focus to work environment quality by demonstrating a specific clinical mechanism through which such efforts will translate into improved safety outcomes.

Second, targeted interventions to enhance nursing surveillance capacity may amplify the safety benefits of improvements to the work environment. Ward practices that strengthen proactive nursing surveillance and that bring registered nurses to the center of decision‐making for patient assessment are critical for hospital safety (Douglas et al. 2024). Operational redesigns that protect dedicated time for patient assessment, minimize non‐essential interruptions during surveillance activities, and enable escalation of early changes and trends in patient status could strengthen the environment‐surveillance‐safety pathway (Henneman 2017). Technology‐enabled nursing assessments that streamline data collection and presentation, provide decision support for pattern recognition, and facilitate rapid communication of concerns may further enhance surveillance effectiveness (Jayousi et al. 2024).

Third, surveillance practices serve as a valuable indicator of process improvement in safety initiatives. Unlike global measures such as safety culture, which reflect complex organizational phenomena and may respond slowly to interventions, surveillance practices are specific, observable, and potentially more responsive to targeted improvements (Bisbey et al. 2021; Flott et al. 2018; Weaver et al. 2013). Regular measurement of surveillance frequency and quality could provide timely feedback for unit‐level learning and system‐level accountability efforts.

### 6.3 Research Strengths and Limitations

This study's strengths include a large, well‐characterized sample of medical‐surgical nurses; validated, theoretically grounded measures of work environment, surveillance, and safety culture; rigorous statistical methodology employing SEM, which incorporates both latent and observed variables, along with bootstrapping to assess indirect effects; and theoretical integration drawing from established frameworks in occupational psychology and healthcare quality.

However, several limitations constrain interpretation and generalizability. The cross‐sectional design precludes causal inference, limiting conclusions to associations rather than causal relationships, despite the theoretical framework supporting mediation analysis in cross‐sectional contexts. Reliance on self‐report measures introduces the possibility of common‐method variance; however, the use of distinct measurement scales and firm theoretical grounding partially mitigates this concern. The surveillance measure, while validated, captures the frequency of core assessment activities rather than the complete cognitive‐behavioral cycle of nursing surveillance, including pattern synthesis, anticipatory reasoning, and escalation decision‐making. In addition, several items within the surveillance scale demonstrated relatively small factor loadings (< 0.4), indicating potential measurement limitations. Generalizability is limited to similar medical‐surgical nursing contexts, and safety culture remains a perceptual outcome that, while important, should ultimately be linked to clinical endpoints such as adverse events and patient outcomes.

These limitations point to several priority directions for future research. Longitudinal and quasi‐experimental designs are crucial for determining whether interventions targeting the work environment or nursing surveillance have causal effects on safety outcomes. Such studies could evaluate specific intervention components, such as staffing improvements, leadership development, and enhanced surveillance capacity, and track their effects over time. Future investigations should also incorporate objective indicators of surveillance quality, including the completeness and timeliness of vital sign assessments, the timeliness of clinical escalations, patterns of rapid response team activations, and rates of unplanned transfers to the intensive care unit. Integrating these objective measures with self‐reported surveillance frequency would yield a more comprehensive assessment of the surveillance process. Finally, linking surveillance practices to objective clinical outcomes, such as preventable adverse events, failure‐to‐rescue rates, and patient mortality, would establish the ultimate validity of surveillance as a safety‐critical process and strengthen the empirical basis for surveillance‐focused interventions.

### 6.4 Policy and Practice Recommendations

The study findings support several recommendations for healthcare policy and practice across organizational, operational, technological, and educational domains. At the organizational level, healthcare systems should prioritize comprehensive work environment improvements, recognizing that such efforts produce safety benefits through multiple pathways, including enhanced nursing surveillance (Peet et al. 2019). Leadership development programs should explicitly address how managerial decisions shape environmental support and influence frontline clinical processes. Operational policies should protect dedicated time for systematic patient assessment, minimize non‐essential interruptions during surveillance activities, and ensure that standardized escalation protocols are routinely developed, reviewed, and enacted so that early changes detected through surveillance reliably prompt timely clinical responses. Technological advancements should be directed toward tools that enhance rather than burden surveillance, with careful attention to workflow integration and usability. Educational initiatives should position surveillance as a core nursing capability, supported by ongoing skill development in systematic physical assessment, pattern recognition, and clinical decision‐making. Finally, quality and safety improvement programs should incorporate surveillance process measures alongside traditional outcome indicators to provide more proximal feedback on safety efforts. At the same time, local practice development approaches can further enable practitioner‐led initiatives that support continuous improvement in ward surveillance practices and safety culture.

## 7 Conclusion

This study establishes nursing surveillance as a mediator of the work environment‐patient safety culture relationship, providing empirical support for a theoretically grounded mechanism linking structural conditions to safety outcomes. This finding is consistent with the multifaceted nature of safety culture and advances our understanding of the specific pathways through which environmental improvements translate into enhanced safety. The results support complementary improvement strategies targeting both structural work environment enhancements and surveillance capacity. This research reinforces the importance of work environment quality as a foundational aspect of patient safety, while identifying surveillance as a specific, actionable process through which organizations can develop more comprehensive approaches to safety improvement. By combining structural investments with targeted process enhancements, organizations can create safer care environments.

## Author Contributions

All authors were involved in the conception and design of the study and the analysis and interpretation of the data, had full access to all of the data in this study and take complete responsibility for the integrity of the data and the accuracy of the data analysis, contributed to the preparation of the manuscript, and approved the manuscript for submission.

## Conflicts of Interest

The authors declare no conflicts of interest.

## Data Availability

To preserve patient privacy and confidentially, the terms of the data use agreement between the study authors and data owners preclude public data sharing. Statistical code for the analysis is available from the study authors on request.
